# Comparison of different delivery systems of DNA vaccination for the induction of protection against tuberculosis in mice and guinea pigs

**DOI:** 10.1186/1479-0556-5-2

**Published:** 2007-01-24

**Authors:** Lúcia de Paula, Célio L Silva, Daniela Carlos, Camila Matias-Peres, Carlos A Sorgi, Edson G Soares, Patrícia RM Souza, Carlos RZ Bladés, Fábio CS Galleti, Vânia LD Bonato, Eduardo DC Gonçalves, Érika VG Silva, Lúcia H Faccioli

**Affiliations:** 1 Departamento de Análises Clínicas, Toxicológicas e Bromatológicas, Faculdade de Ciências Farmacêuticas de Ribeirão Preto, Universidade de São Paulo, Av. do Café s/n, 14040-903, Ribeirão Preto, SP, Brasil; 2 NPT – Núcleo de Pesquisas em Tuberculose – Departamento de Bioquímica e Imunologia, Faculdade de Medicina de Ribeirão Preto, Universidade de São Paulo, Av. Bandeirantes, 3900, 14049-900, Ribeirão Preto, SP, Brasil; 3 Departamento de Patologia, Faculdade de Medicina de Ribeirão Preto, Universidade de São Paulo, Av. Bandeirantes, 3900, 14049-900, Ribeirão Preto, SP, Brasil; 4 Farmacore Biotecnologia Ltda, Rua dos Técnicos s/n, Campus da USP – Ribeirão Preto, SP, Brasil

## Abstract

The great challenges for researchers working in the field of vaccinology are optimizing DNA vaccines for use in humans or large animals and creating effective single-dose vaccines using appropriated controlled delivery systems. Plasmid DNA encoding the heat-shock protein 65 (hsp65) (DNAhsp65) has been shown to induce protective and therapeutic immune responses in a murine model of tuberculosis (TB). Despite the success of naked DNAhsp65-based vaccine to protect mice against TB, it requires multiple doses of high amounts of DNA for effective immunization. In order to optimize this DNA vaccine and simplify the vaccination schedule, we coencapsulated DNAhsp65 and the adjuvant trehalose dimycolate (TDM) into biodegradable poly (DL-lactide-co-glycolide) (PLGA) microspheres for a single dose administration. Moreover, a single-shot prime-boost vaccine formulation based on a mixture of two different PLGA microspheres, presenting faster and slower release of, respectively, DNAhsp65 and the recombinant hsp65 protein was also developed. These formulations were tested in mice as well as in guinea pigs by comparison with the efficacy and toxicity induced by the naked DNA preparation or BCG. The single-shot prime-boost formulation clearly presented good efficacy and diminished lung pathology in both mice and guinea pigs.

## Background

Tuberculosis (TB) still remains a major health problem affecting millions of people worldwide [1]. The only TB vaccine currently available is *Mycobacterium bovis *BCG. However, the efficacy of BCG still remains controversial, especially against pulmonary TB in young adults, and development of a better vaccine is urgently required to counteract the global threat of TB [2-4]. Over the past 20 years, the technology of vaccine development has changed radically. The strategy of using the pathogen itself has given way to creating alternate forms of antigens (such as genes encoding specific antigens), new adjuvants and new delivery systems, as well as employing the recently devised prime-boost concept [5-8]. Thus, several strategies have been employed for generation and evaluation of new TB vaccines. Recombinant BCG strains, DNA-based vaccines, live attenuated *M. tuberculosis *vaccines and subunit vaccines formulated with novel adjuvants have shown promise in preclinical animal challenge models. The ability of DNA vaccines to elicit Th1 biased CD4+ responses and strong CTL responses make them particularly attractive weapon against *M. tuberculosis *infection [9,10].

Experimental data collected over several years by our group showed that the DNA vaccine codifying the 65 kDa heat shock protein from *M. leprae *(DNAhsp65) presented a prophylactic and therapeutic effect in a murine model of TB [11-13]. Although the prophylactic effect demonstrated initially with this vaccine was equal from live BCG vaccine in mice and the feature of this protection were associated with the presence of CD8+/CD44^hi ^IFN-γ-producing/cytotoxic cells [14] there was a necessity to optimize the vaccine formulation in order to improve efficacy and diminish possible toxicity. The literature on plasmid DNA in vaccination suggests that four doses of naked DNA injected intramuscularly might not be sufficient for the generation of protective humoral and cellular immune responses against infectious diseases in large animals [15-18]. One of the pharmaceutical measures to improve DNA vaccines has been to ameliorate the uptake of DNA into professional antigen-presenting cells by using DNA entrapped into polymeric particles. Biodegradable poly (lactic-co-glycolic acid) microspheres (PLGA) represent an attractive candidate for vaccine delivery [19]. Therefore, as an alternative strategy to confer long-lasting protection against TB in mice and guinea pigs, we evaluated the encapsulation of the hsp65-DNA and rhsp65 into biodegradable PLGA microspheres that have the potential to release the antigen in a sustained fashion. Due to its ability to induce the secretion of cytokines in a Th1 pattern of immune response [11], trehalose dimycolate (TDM), a glycolipid from the *Mycobacterium *cell wall, was included in the formulations as an adjuvant. Moreover, a more recently devised tool for generating a protective and long-lasting immune response involves combining different vehicles carrying the same immunogen in heterologous prime-boost protocols [20]. These prime-boost vaccination strategies consist of using two different vaccines, each encoding the same antigen, administered some weeks apart. In the prevention of TB, the prime-boost strategy of combining DNA priming and boosting with BCG or recombinant proteins has been evaluated by various authors [21,22]. Such protocols typically require more than one high amount of DNA dose for priming, followed by a booster with live vectors, thereby necessitating the use of large quantities of DNA. Taking into account these factors we also evaluated both in mice and guinea pigs the use of a new concept in vaccine formulation based on a mixture of two different PLGA microspheres, presenting faster and slower release of, respectively, DNA encoding hsp65 and the recombinant hsp65 protein [20]. Our aim was to achieve DNA priming and protein boosting after a single-dose vaccination.

## Materials and methods

### Animals

Outbred Female Hartley guinea pigs weighing 300–350 g and young adult BALB/c mice were obtained from the animal facilities of the campus of Ribeirão Preto, Universidade de São Paulo, and were maintained under standard laboratory conditions. Infected animals were kept in biohazard facilities, housed in cages within a laminar flow safety enclosure. All experiments were approved and conducted in accordance with guidelines of the Animal Care Committee of the University.

### Plasmid derivation

The construction of a pVAX plasmid (Invitrogen) containing the cytomegalovirus (CMV) promoter and a cDNA encoding the HSP65 gene for *M. leprae *(pVAX-HSP65) has been previously described [23]. The vector without the *hsp65 *gene was used as control. DH5α *Escherichia coli *transformed with plasmid pVAX or the plasmid containing the *hsp65 *gene (DNAhsp65) was cultured in LB liquid medium (Gibco-BRL) containing kanamycin (100 μg/mL). Plasmid DNA was obtained as described in the EndoFree plasmid purification handbook (Qiagen, Ltd., Crawley, UK). Spectrophotometric analysis using Gene Quant II apparatus (Pharmacia Biotech, Buckinghamshire, UK) revealed the 260/280 nm ratios to be ≥ 1.80. The purity of DNA preparations was confirmed on the 1% agarose gel.

### Recombinant hsp65 protein

*E. coli *BL21 transformed with the plasmid containing the mycobacterial hsp65 gene was cultured in LB medium containing ampicillin (100 μg/μl). The bacterial growing was monitored by spectrophotometry in a Shimadzu UV-1650 spectrophotometer. When the OD reached the value of 0.6, the culture was induced with 0.1 M of IPTG (Gibco, BRL, Gaithersburg, MD, USA) and incubated at 30°C under agitation for 4 h. Protein purification was done as previously described [24].

### Microspheres preparation

Microspheres were obtained by the double emulsion/solvent evaporation technique as previously described [25]. Briefly, 30 ml dichloromethane solution containing 400 mg of polymer PLGA 50:50 or PLGA 75:25 (Resomerfrom Boehringer Ingelheim, Ingelheim, Germany) and 0.5 mg of TDM (Sigma, St Louis, USA) was emulsified with 0.3 ml of an inner aqueous phase containing 5 mg of DNA (DNAhsp65 or DNAv) or 1 mg of recombinant hsp65 protein using a T25 Ultraturrax homogenizer (IKA – Labortechnik, Germany) to produce a primary water-in-oil emulsion. This emulsion was then mixed with 100 ml of an external aqueous phase containing 1–3 % poly vinyl alcohol (Mowiol 40–88, Aldrich Chemicals, Wankee, WI, USA) as surfactant, to form a stable water-in-oil in-water emulsion. The mixture was stirred for 6 h with a RW20 IKA homogenizer for solvent evaporation. Microspheres were collected and washed three times with sterile water, freeze-dried and stored at 4°C.

### Particle diameter analysis, rate of DNA and protein encapsulation, endotoxin levels, and kinetics of DNA and protein release

Particle diameter distribution was evaluated by laser diffractometry in a Shimadzu Sald 2164 apparatus (Shimadzu, Japan). Results are expressed as median value of diameter distribution. Plasmid encapsulation rate was determined adapted from Barman et al. [26]. Briefly, 10 mg of microspheres were ressuspended in 0.2 ml of TE buffer and 500 μl of chloroform were added to the suspension. The mixture was maintained under agitation for 60 min. The sample was centrifuged at 14,000 rpm for 5 min and the supernatant was separated for analysis. The amount of DNA was determined as described before using the Gene Quant II. The protein encapsulation rate was determined after addition of 0.2 ml of acetonitrile. The sample was incubated in an ultrasound bath for 15 min allowing the complete solubilization of microspheres and it was followed by addition of water (1:1). The protein content was assayed by using the Comassie reagent (Pierce, Rockford, IL, USA). The protein concentration was determined at 600 nm using an ELISA reader (960, Metertech). The kinectics of protein release from microspheres was evaluated by ressuspending 30 mg of protein-loaded microspheres in 3 ml of PBS containing sodium azide (0.05% w/v). The suspension was maintained at 37°C under constant agitation at 200 rpm. In pre-established time intervals, samples of the supernatant (0.1 ml) were collected and replaced with fresh buffer. The protein concentration in the supernatant was determined by using the Comassie Reagent as previously described [19]. The endotoxin detection in formulations was made by *Limulus *Amebocyte Lysate test (LAL test, QCL-1000, Bio Whittaker, CAMBREX). For this purpose microspheres were ressuspended in PBS and the suspension was maintained in vortex until the complete homogenization.

### Immunization procedures

Immunization either in mice or guinea pigs was by one of the following treatments, and five to ten animals were used in each group. For naked DNA vaccination, the plasmid DNAhsp65 was administered by intramuscular injection of 100 μg DNA in saline into each quadriceps muscle on three occasions at 2-week intervals (total dose of 300 μg of plasmid). Additional control animals received saline or control vector (DNAv) by using the same amount and schedule of treatments. BCG (Pasteur strain) was given as a single subcutaneous injection of about 10^5 ^live bacteria in 50 μl saline. Animals received a single-dose of an intramuscular injection of 2.5 mg of microspheres in 50 μl saline into each quadriceps muscle. Two microspheres formulations were evaluated: DNAhsp65/TDM-loaded PLGA 50:50 microspheres (Me-DNAhsp65/TDM) and a mixture (1:1 w/w) of DNAhsp65/TDM-loaded PLGA 50:50 microspheres and recombinant hsp65 protein/TDM-loaded PLGA 75:25 microspheres (Me-Prime/boost). Additional control animals received DNAvector/TDM-loaded PLGA 50:50 microspheres (Me-control).

### Challenge infection of immunized animals

Guinea pigs and mice were challenged by intratracheal route with 10^5 ^colony-forming units (CFU) of *M. tuberculosis *H37Rv, 30 days after the last immunization. Animals were killed 30 days after infection and the number of live bacteria in the lungs was determined as CFU by plating 10-fold serial dilutions of homogenized tissue on Middlebrook 7H11 agar (Difco), counting colonies after 21 days and results expressed as log_10 _CFU/g lung tissue.

### Histology

The upper left lobe of each animal was fixed in 10% formalin, embedded in paraffin blocks, prepared routinely, then sectioned for light microscopy. Sections (5 μm each) were stained either with haematoxylin & eosin method. Cellular infiltrate in lung parenquima was analysed by morphometrical measures. Results were expressed as percent of cellular infiltrate in lung parenchyma of animals previously vaccinated with different formulations and challenged with *M. tuberculosis*.

### Statistical analysis

Results were expressed as mean (±) SD. Significance of difference among groups was calculated by Student's *t *tests.

## Results

### Encapsulation efficiency and physical characteristics of DNAhsp65/TDM-loaded microspheres

Plasmid DNA was incorporated into PLGA microspheres by the double emulsion/solvent evaporation method. Encapsulation efficiency varied from 30 to 50% for DNA and around 70% for protein. Table 1 shows the amount of entrapped DNA and protein in the different formulations. In this work, particles were designed to have diameter smaller than 10 μm. Particles encapsulating DNA were greater in diameter than microspheres encapsulating protein (Table 1). The association of TDM with protein or DNA in microspheres did not change the diameter or loading rate after encapsulation. Microspheres population presents characteristic Gaussian distribution of diameter. The microspheres formulations were also assayed for detection of endotoxin activity using the *Limulus *amebocyte assay (LAL test). We showed in the Table 1 that endotoxin activity in all microsphere formulations was lower than 0.4 EU/mg. According to the European Pharmacopoeia the safety level for endovenous administration is 5 EU/Kg/hour that corresponds to 0.1 EU per mouse (20 g) per hour.

**Table 1:** Median values of diameter distribution, encapsulation rate, and endotoxin levels in each formulation

**Formulation**	**Composition**	**Average diameter (μm)**	**Encapsulation rate** ** (μg/mg of microspheres)** **DNA – Protein**		**Endotoxin level (UE/mg)**
Me-Control	- 100% of DNAv plus TDM-loaded PLGA 50:50 microspheres	4.0	4.11	-	0.011
Me-DNAhsp65/TDM	- 100% of DNAhsp65 plus TDM-loaded PLGA 50:50 microspheres	3.7	4.97	-	0.028
Me-Prime/boost	A mixture of PLGA microspheres containing:				
	- 50% of DNAhsp65 plus TDM-loaded PLGA 50:50 microspheres	3.5	4.90	-	0.030
	- 50% rHsp65 protein plus TDM-loaded PLGA 75:25 microspheres	2.4	-	1.70	0.053

### In vitro hsp65 protein, TDM and DNA release profiles from PLGA microspheres

**Figure 1:**
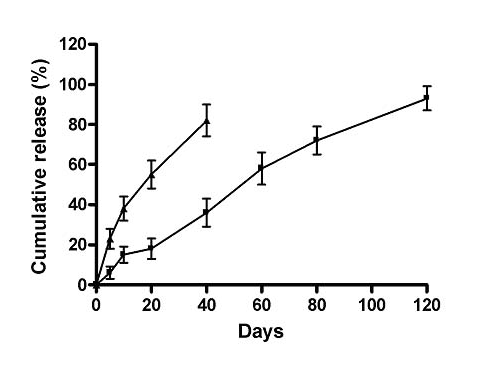
*In vitro *release profile of DNAhsp65 (▲) and recombinant hsp65 protein (■) encapsulated into PLGA derived microspheres. PLGA derived microspheres containing DNA or rHsp65 were ressuspended in PBS and maintained at 37°C under constant agitation. In pre-established time intervals, samples of the supernatant were collected and replaced with fresh buffer. The DNA or protein concentration in the supernatants were determined and represented as a percentage o cumulative release. Results are shown as mean μg ± SD from groups of five samples.

The *in vitro *release profiles of recombinant hsp65 protein entrapped into PLGA microspheres were evaluated *in vitro *for over 120 days. The results showed that microspheres released all the encapsulated protein in a 90-day interval. Most of the protein was released after 50 days (Figure 1). The Me-DNAhsp65/TDM formulation released around 80% of their DNA or TDM (not shown) load after 20 days.

### Protection against *M. tuberculosis *replication in lungs of vaccinated animals

As shown in Table 2, a single dose of Me-DNAhsp65/TDM formulation was able to protect mice as well as guinea pigs against *M. tuberculosis *as efficiently as three doses of naked DNAhsp65. There was a significant reduction in the number of bacterial burden compared to control mice, which is similar to the reduction provided by BCG in both animal models, which therefore can be considered as good protection. The same level of protection as described for Me-DNAhsp65/TDM was observed in mice and guinea pigs vaccinated with Me-Prime/boost formulation in a single dose.

**Table 2:** Bacterial replication in lungs from mice and guinea pigs vaccinated and challenged with *M. tuberculosis*

**Vaccine formulations**^**a**^	**Mice**^**b**^	**Guine-pigs**^**b**^
No vaccination	6.12 ± 0.40	5.43 ± 0.38
BCG	3.25 ± 0.38*	3.80 ± 0.24*
DNAv (naked DNA)	6.02 ± 0.35	5.68 ± 0.29
DNAhsp65 (naked DNA)	4.76 ± 0.26*	4.90 ± 0.25*
Me-DNAhsp65/TDM	4.55 ± 0.28*	4.12 ± 0.25*
Me-Prime/boost	4.21 ± 0.30*	4.54 ± 0.27*

^**a**^Mice and guinea pigs (5–10 animals per group) were immunized by the following schedule: PBS (three intramuscular injection at 2-week intervals); DNA-hsp65 (three intramuscular injection of 100 ug DNA plasmid encoding *M. leprae *hsp65 gene at 2-week intervals); DNAv (plasmid DNA without the hsp65 gene administered at the same scheme for DNAhsp65); BCG (single intradermic injection of about 10^5 ^live bacteria in 50 ml saline); Me-DNAhsp65/TDM-loaded microspheres (single intramuscular injection of DNAhsp65 plus TDM-loaded PLGA 50:50 microspheres); Me-Prime/boost (single intramuscular injection of a mixture of PLGA microspheres as described in Table 1). Guinea pigs and mice were challenged by intratracheal route with 10^5 ^CFU of *M. tuberculosis *H37Rv, 30 days after the last immunization.

^**b**^Animals were killed 30 days after infection and the number of live bacteria in the lungs was determined as mean number of CFU ± SD (log10 values)/g lung tissue.

* Indicate that the effects of vaccination were significant compared with data from animals not vaccinated and challenged with *M. tuberculosis *(Student's t-tests, *P *< 0.05).

### Histological analysis of lungs from mice and guinea pigs vaccinated and challenged with virulent strain of *M. tuberculosis*

Thirty days after challenge, animals were sacrificed and lungs were processed for histological analysis. Non-vaccinated and challenged animals were used as control groups and the results are illustrated in Table 3. We observed that in the mice control group (no vaccinated and *M. tuberculosis*-infected) around 70% of lung parenchyma was compromised with granuloma formation widely distributed, presenting cellular infiltrate containing mainly lymphocytes, with few macrophages and without neutrophils. Mice or guinea pigs injected with DNAhsp65 (naked DNA) presented a very low and similar compromising of lungs. However, there were few and small granulomas in this group with cell infiltrate composed by lymphocyte, macrophages, neutrophils and plasmocytes without of necrotic areas. The group vaccinated with Me-DNAhsp65/TDM formulation presented lower lung compromising with lymphocytes and macrophage infiltration, as well as few neutrophilic infiltrations (Table 3). Me-Prime/boost vaccinated mice also presented lower lung compromising with cellular infiltration characterized by high amount of macrophages. In some areas tissue showed lymphocytes concentrated around bronchus.

**Table 3:** Percent of cellular infiltrate in lung parenchyma of animals vaccinated and challenged with *M. tuberculosis*

**Formulations**^**a**^	**Mice**^**b**^	**Guine-pigs**^**b**^
No vaccination	74 ± 8	69 ± 9
BCG	18 ± 5*	24 ± 4*
DNAv (naked DNA)	72 ± 9	65 ± 7
DNAhsp65 (naked DNA)	38 ± 7*	42 ± 6*
Me-DNAhsp65/TDM	41 ± 6*	40 ± 7*
Me-Prime/boost	37 ± 5*	38 ± 6*

^**a**^Mice and guinea pigs were immunized as described in Table 1.

^**b**^Animals were killed 30 days after infection and the lung cellular infiltrates were measured and expressed as percent of cellular infiltrate in lung parenchyma.

* Indicate that the effects of vaccination were highly significant compared with data from animals not vaccinated and challenged with *M. tuberculosis *(Student's t-tests, *P *< 0.001).

## Discussion

The high incidence of TB around the world and the inability of BCG to protect certain populations clearly indicate that an improved vaccine against TB is needed. Currently, many vaccines are under development and there is a desire to simplify vaccination schedules by decreasing the number of doses. With this purpose, various substances have been added to vaccines and certain formulations have been devised in an attempt to render vaccines more effective [27]. Despite of success of naked DNAhsp65-based vaccine to protect mice against TB, it requires multiple doses of high amount of plasmid for effective immunization, which could lead to an exacerbated inflammatory reaction in the lungs of challenged mice or guinea pigs. To optimize this DNA vaccine, we used an approach where adjuvants with targeting and immunostimulatory properties prepared by microencapsulation techniques are administered in conjunction with the DNA-encoding antigen. BALB/c mice immunized with a single dose of Me-DNAhsp65/TDM-loaded microspheres produced high levels of IgG2a subtype antibody and high amounts of IFN-γ in mice as previously described [20,24]. Here we show that Me-DNAhsp65/TDM-loaded microspheres were also able to confer protection as effective as that attained after three doses of naked DNA administration either in mice or guinea pigs. This new formulation also allowed a ten-fold reduction in the DNA dose when compared to naked DNA as well as a significant reduction in the cellular infiltrate in the lung parenchyma of mice and guinea pigs. Thus, this combination of DNA vaccine and adjuvants with immunomodulatory and carrier properties holds the potential for an improved vaccine against TB. PLGA biodegradable microspheres also have the potential to act as mediators of DNA transfection targeted to phagocytic cells such as macrophages or dendritic cells, and to protect against biological degradation by nucleases [28,29]. We previously show that DNAhsp65-loaded microsphere without adjuvant was unable to protect mice against challenge [23]. Thus, the entrapment of DNA plus an immunostimulant compound into PLGA microspheres could be an interesting strategy for vaccine formulation. The adjuvant effect of purified TDM on immune response has been recognized long ago [30]. The immunostimulatory activities made TDM an attractive candidate for adjuvant use in vaccine formulation. Moreover, the polymer has an established clinical safety record and its slow degradation permits sustained delivery of antigen [31]. Hence, if the quality of the immunity is dependent on antigen persistency, or if compliance is compromised due to socio-economic or demographic circumstances, PLGA-like microspheres offer a potential advantage for the vaccines.

A more recently devised tool for generating a protective and long-lasting immune response involves combining different vehicles carrying the same immunogen in heterologous prime-boost protocols [20]. These prime-boost vaccination strategies consist of using two different vaccines, each encoding the same antigen, administered some weeks apart. Most prime-boost protocols currently under evaluation include priming with DNA and boosting with viral vectors [32,33]. This strategy resurrects questions concerning the safety of using live attenuated viruses that have been replaced by subunit or DNA vaccines alone. In the prevention of TB, the prime-boost strategy of combining DNA priming and boosting with BCG or recombinant proteins has been evaluated by various authors [17,19]. Such protocols typically require more than one DNA dose for priming, followed by a booster with live vectors, thereby necessitating the use of large quantities of DNA. As shown above, the encapsulation of antigen into PLGA microspheres allows the development of controlled-release delivery systems, in which the release profile of the encapsulated material can be tailored to specific purposes. Taking advantage of this fact, we evaluated the use of a vaccine formulation based on a mixture of two different PLGA microspheres, presenting faster and slower release of, respectively, DNA-hsp65 and the rhsp65 (Figure 1). Our aim was to achieve DNA priming and protein boosting after a single-dose vaccination. We demonstrated previously in mice [20], that the Me-Prime/boost formulation induced high levels of anti-hsp65 antibodies and IFN-γ, which remained high 90 days after vaccination, whereas the Me-DNAhsp65/TDM formulation was unable to sustain antibody levels in the same fashion. Here we show that mice or guinea pigs challenged with a virulent strain of *M. tuberculosis *30 days after vaccination, we observed significantly lower numbers of CFUs in the lungs of those vaccinated with Me-Prime/boost formulation than in the lungs of the controls. Moreover, we showed that the infection remained under control and the lung parenchyma unaffected only in the group immunized with the Me-Prime/boost formulation. These data suggest that Me-Prime/boost is a formulation capable of sustaining the protective response in mice and guinea pigs. Therefore, using biodegradable microspheres in a single dose seems to be a promising strategy for stimulating long-lasting immune responses in large animals.

In this study, we set out to overcome significant obstacles currently faced in for the field of DNA vaccine development. We described, for the first time, the development of a single dose/prime-boost DNA vaccine formulation for immunizing mice and guinea pigs against mycobacterial challenge. This new technology allows radically different approaches to the problems of immunization with DNA vaccines. Furthermore, using combinations of vaccines and alternative routes of administration will allow researchers to customize vaccination programs. Moreover, this new technique may increase veterinarian and patient acceptance of vaccination by reducing the number of injections and avoiding the use of boosters containing live vectors. Using this technology, vaccinologists could develop many DNA vaccines that would induce specific forms of immunity, access new routes of delivery, provide increased safety when necessary, be more stable and lower costs. We believe that this strategy can be applied to vaccines for humans and to other veterinary vaccines, thereby having a tremendous impact on the control of infectious diseases in humans and in large animals.

## Authors' contributions

Thirteen researchers participated in this study. LP and CLS are the principal investigators in this study. DC, CMP, CAS and EVGS participated in the experiments accomphished with guinea pigs. EGS helped with histological analysis. Experiments involving mice were done by PRMS, FCSG, EDCG, VLDB and CRZB in the laboratory of CLS and the Company Farmacore Biotecnologia Ltda, who also shared their expertise in the DNA vaccine. The majority of the research was done in the laboratory of LHF who coordinated the projected and provided critical input and assistance.
